# First reported human case of isolation of *Vagococcus fluvialis* from the urine of a former zoo clerk in Japan: a case report

**DOI:** 10.1186/s12879-024-09193-4

**Published:** 2024-03-21

**Authors:** Hiroyuki Kitano, Hiroki Kitagawa, Kayoko Tadera, Kohei Saito, Yuki Kohada, Kenshiro Takemoto, Kohei Kobatake, Yohei Sekino, Keisuke Hieda, Hiroki Ohge, Nobuyuki Hinata

**Affiliations:** 1 Department of Urology, Graduate School of Biomedical and Health Sciences, Hiroshima University, 734-8551 Hiroshima, Japan; 2 Department of Infectious Diseases, Hiroshima University Hospital, 734-8551 Hiroshima, Japan; 3 Department of Clinical Practice and Support, Hiroshima University Hospital, 734-8551 Hiroshima, Japan

**Keywords:** *Vagococcus fluvialis*, Urinary tract infection, Zoo clerk

## Abstract

**Background:**

Vagococcal infections are extremely rare in humans. There are limited studies on the optimal methods for identification, antimicrobial susceptibility testing, and clinical manifestations of vagococcal infections. Herein, we report a patient with a urinary tract infection who had *Vagococcus fluvialis* in the urine.

**Case presentation:**

An 84-year-old man presented to our urology department with a fever that had persisted for several days. He previously worked as a zoo clerk. The patient underwent a left nephroureterectomy for ureteral cancer 5 years ago, and total cystectomy and right cutaneous ureterostomy for muscle-invasive bladder cancer 1 year prior. He was empirically treated with 500 mg of levofloxacin intravenously every 24 h for the urinary tract infection. *V. fluvialis* was detected in his urine samples and *Pseudomonas aeruginosa* was detected in his urine and blood samples. Two bacterial species were identified using matrix-assisted laser desorption/ionization time-of-flight mass spectrometry. He was administered intravenous levofloxacin for approximately 1 week, followed by oral levofloxacin for another week, after which the infections were eradicated.

**Conclusions:**

To the best of our knowledge, this is the first report of *V. fluvialis* detected in human urine in Japan. *Vagococcus* spp. is commonly isolated from fish or animals, and based on the patient’s work history, it is possible that the patient was a carrier because of transmission from animals.

## Background

*Vagococcus* spp. is a genus of gram-positive, catalase-negative, facultative anaerobic cocci comprising 14 species [1]. *Vagococcus fluvialis* was first isolated in 1974 from chicken feces and river water; initially, these isolates were identified as members of the *Lactococcus* spp. However, in 1989, these isolates were classified as a new genus, *Vagococcus* spp, using 16 S rRNA sequencing [2]. Since then, many of these species have been isolated from animals; however, in 1997 *V. fluvialis* was first isolated from human blood, peritoneal fluid, and wounds [3]. The LPSN (List of Prokaryotic names with Standing in Nomenclature) database (Accessed on February 24, 2024, https://www.bacterio.net/) comprises genetic data concerning nine strains of *Vagococcus fluvialis*. Although human infectivity has been reported, the number of reports is very small. To the best of our knowledge, this is the first report on *V. fluvialis* isolated from human urine in Japan.

## Case presentation

An 84-year-old man presented to the Department of Urology in Hiroshima University Hospital with a high fever that had persisted for several days. He was a retired clerk in a zoo.

Five years prior to presentation, he underwent total nephroureterectomy for left ureteral cancer, and 1 year prior to presentation, he had undergone total cystectomy and right ureterocutaneostomy. A ureteral stent was inserted during ureterocutaneostomy and replaced every month. When he presented to our hospital, the stent had spontaneously fallen out of his ureterocutaneostomy. The patient was admitted to the hospital where he developed fever (39.0 °C) and tachycardia (heart rate 129 beats/min), with a blood pressure of 166/115 mmHg. The urinary pH was determined to be 6.5, accompanied by the identification of 20 to 29 erythrocytes per high-power field (HPF) and 50 to 99 leukocytes per HPF. Adverse results were absent for urinary glucose and protein. A complete blood count revealed leukocytosis (1.39 × 10^9^ cells/L), a thrombocytopenia (151 × 10^9^ cells/L). No deviations in hemoglobin levels, hepatic functionality, or electrolyte homeostasis were detected. Acute kidney injury (serum creatinine 2.38 mg/dl) was detected. C-reactive protein level was elevated (2.75 mg/dL). Computed tomography revealed right hydronephrosis and inflammatory changes in perirenal fat. The patient was diagnosed with right pyelonephritis and hydronephrosis. Pelvic urine and blood culture samples were submitted for analyses.

*V. fluvialis* and *Pseudomonas aeruginosa* were cultured from pelvic urine samples and bacterial concentrations of 10^5^ colony-forming units per milliliter (CFU/ml) were identified in each bacterial species. Only *P. aeruginosa* was cultured from blood culture.

**Fig. 1:**
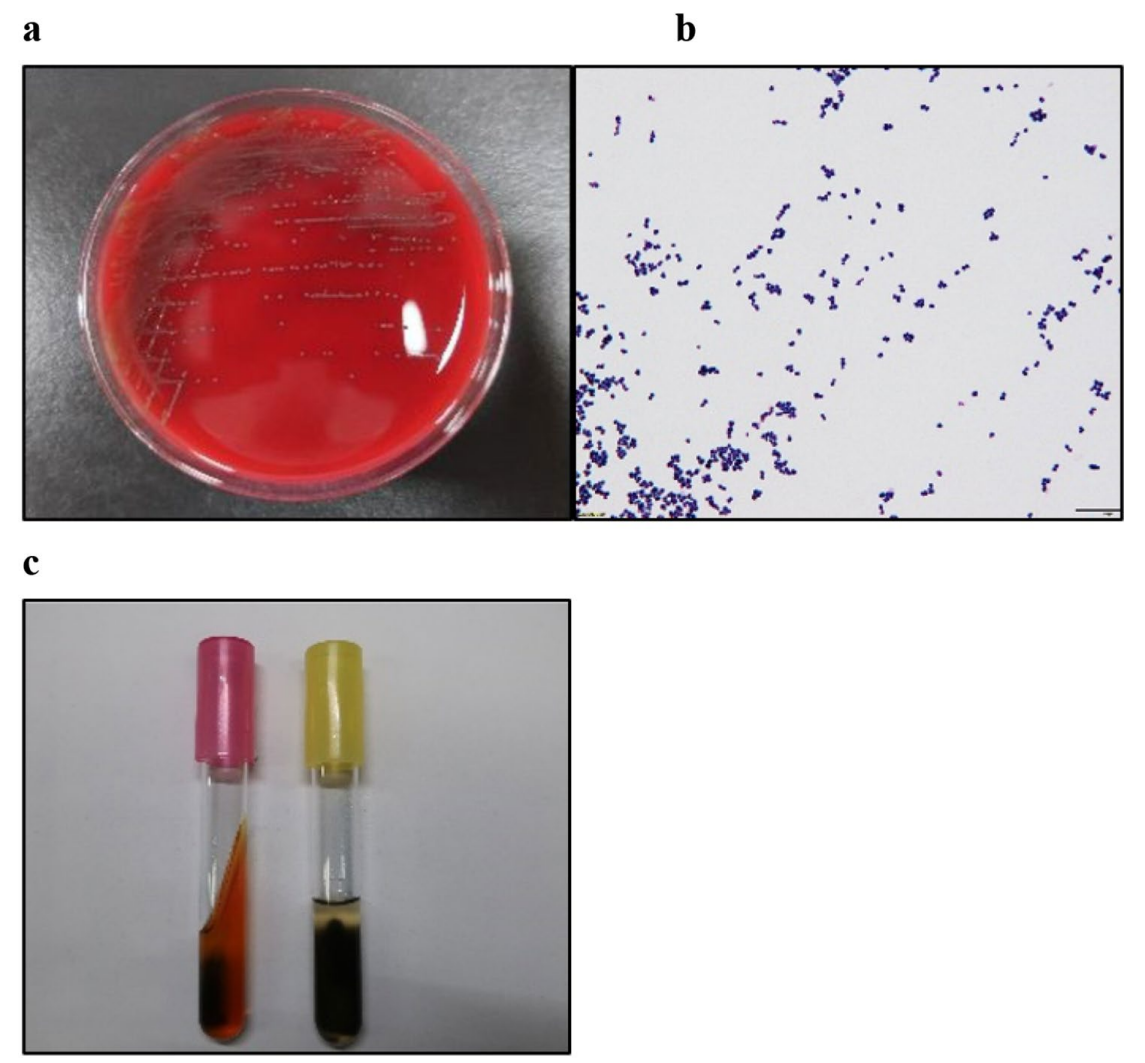
Colony and gram staining and motility of *Vagococcus fluvialis.* (a) Steak-purified culture of *Vagococcus fluvialis* on 5% sheep blood agar. (b) Gram staining of vagococci. Gram-positive strain of cocci in groups of 2 to 5 cells. (c) Triple-sugar iron and sulfide indole motility media changed black at 36 °C

A matrix-assisted laser desorption ionization time-of-flight mass spectrometry (MALDI-TOF MS) analysis was performed to identify these pathogens using a BD MALDI Biotyper Sirius system (Becton, Dickinson and Company, Franklin Lakes, NJ, USA) with MBT Compass 4.1, from the MBT Compass library: Ver. 9.0.0.0 (8468MSPs) (Bruker Daltonik GmbH, Bremen, Germany) database. The MALDI-TOF MS analysis using pure culture colonies was performed by direct transfer methods. Positive urine extracts were cultured on 5% sheep blood agar (Eiken Chemical Co., Ltd., Tokyo, Japan) at 36 °C. The colonies of *V. fluvialis* were not small, and gram staining showed clusters of 2–5 cells (Fig. 1a and b). *V. fluvialis* produced H_2_S on triple-sugar iron (Kyokuto Pharmaceutical Co., Tokyo, Japan) and sulfide indole motility (Kyokuto Pharmaceutical Co., Tokyo, Japan) media (Fig. 1c) at 36 °C. The MALDI-TOF MS analysis of these colonies identified *V. fluvialis* with a score of 2.24. The strain exhibited concordance with eight *V. fluvialis* strains cataloged in the MALDI Biotyper Sirius system, achieving a score of at least 2.

To determine the bacterial susceptibility to antibiotics, we measured the minimum inhibitory concentration (MIC) of antibiotics against the strain of *V. fluvialis* isolated via the broth microdilution method using IA40 MIC-i with Dry Plates (Eiken Chemical) referring to the previous report [3]. The MICs (µg/mL) were as follows: penicillin G, 0.5; ampicillin, 0.25; cephazoline, > 2; ampicillin/sulbactam, 0.25; imipenem, 0.25; ceftriaxone, > 2; vancomycin, > 1; levofloxacin, > 4; ciprofloxacin, > 2; trimethoprim/sulfamethoxazole, 5; and clindamycin, > 1.

Treatment was initiated with levofloxacin (LVFX) based on the exclusive identification of *Pseudomonas aeruginosa* in the bloodstream, pending consideration of antimicrobial susceptibility results for *V. fluvialis*, a process that may take several days. LVFX (500 mg/body every 24 h) was initiated on admission for 6 days intravenously and switched to oral administration of LVFX (500 mg/body every 24 h). After a total of 14 days of treatment, the patient recovered and was transferred to another hospital to improve activities of daily living.

## Discussion and conclusions

To the best of our knowledge, this is the first case report of *V. fluvialis* detected in human urine in Japan. *V. fluviali*s was initially recognized in the year 1989 due to its resemblance in attributes to those exhibited by Enterococcus and Lactococcus. Consequently, discernment between these microbial species solely founded upon morphological traits remains unfeasible [3].. *V. fluvialis* is a species of lactic acid bacteria found free-living in rivers and seawater [4] and associated with hosts, such as pigs, cattle, cats, horses, fish, and marine sponges [2, 4, 5–7]. To date, only 14 cases of Vagococcal infections in humans have been reported [8, 9]. In these patient reports, *V. fluvialis* was detected in wounds of skin and soft tissue infection, blood of infective endocarditis, peritoneal fluid of peritonitis, cerebrospinal fluid of meningitis, root canal of endodontic infection, and urine either by 16 S rRNA and MALDI-TOF-MS, or each alone [8, 9]. Only one case of Vagococcal infection was detected in a skin pressure ulcer and blood specimen, suggesting that the portal for *V. fluvialis* entry may be the skin [8]. In our patient, *V. fluvialis* was detected in the urine. However, it was not detected in the blood, as was *P. aeruginosa*. The direct causative pathogen of his infection may have been *P. aeruginosa*. The pathogenicity of *V. fluvialis* in humans is not clear, although it has been found to infect human urine in the present patient. *V. fluvialis* is detected in animals and fish, although the predominant route of transmission for this pathogen remains to be determined [4–6]. However, our patient was not an animal keeper and he just worked in a zoo; this new finding suggests that *V. fluvialis* can be transmitted from animals and fish to humans.

Little is known regarding the susceptibility of *V. fluvialis* to antimicrobials. *Vagococcus* species are susceptible to ampicillin, cefotaxime, trimethoprim/sulfamethoxazole, vancomycin, and linezolid, but resistant to clindamycin, levofloxacin, and ofloxacin [3, 8, 9]. The strain isolated from our patient had a low MIC for penicillin, ampicillin, ampicillin/sulbactam, imipenem, and trimethoprim/sulfamethoxazole.

There is no established method for identifying *V. fluvialis*; we used MALDI-TOF MS to identify this strain. As *V. fluvialis* is morphologically similar to *Enterococcus spp.*, 16 S rRNA or MALDI-TOF MS may be useful, as reported previously [10].

In conclusion, we reported one case of *V. fluvialis* infection detected in human urine in Japan, which, to the best of our knowledge, is the second case in the world. If *V. fluvialis* is detected in human urine along with a urinary tract infection, it may be necessary to ask about the patient’s work history.

## Data Availability

The datasets generated and/or analyzed during the current study are available from the corresponding author on reasonable request.

## Abbreviations

*MALDI-TOF MS*: Matrix-assisted laser desorption ionization time-of-flight mass spectrometry
*MIC*: Minimum inhibitory concentration
*LVFX*: Levofloxacin
